# Unusual cause of small bowel obstruction in a liver transplant recipient: a case report and focused literature review

**DOI:** 10.3389/fsurg.2026.1824834

**Published:** 2026-06-08

**Authors:** Anastasia Sotiropoulou, David Martin, Julien Vionnet, Amaniel Kefleyesus

**Affiliations:** 1 Faculty of Medicine, National and Kapodistrian University of Athens, Athens, Greece; 2 Department of Visceral Surgery, Lausanne University Hospital (CHUV) and University of Lausanne, Lausanne, Switzerland; 3 Transplantation Center, Service of Immunology and Allergy, Service of Gastroenterology and Hepatology, Lausanne University Hospital (CHUV) and University of Lausanne, Lausanne, Switzerland

**Keywords:** bezoar, case report, indocyanine green fluorescence, liver transplantation, phytobezoar, small bowel obstruction

## Abstract

**Background:**

Small bowel obstruction (SBO) is a common surgical emergency. In transplant recipients, atypical etiologies may delay diagnosis and lead to suboptimal management.

**Methods:**

We report a case of phytobezoar-induced SBO in a liver transplant recipient and conducted a focused narrative literature review (PubMed/MEDLINE, Embase, Cochrane Library, Google Scholar, OpenEvidence, Litmaps) of adult transplant patients with mechanical SBO reported up to January 2026.

**Results:**

A 60-year-old man with two prior liver transplantations (2012, 2022) presented with acute abdominal pain and vomiting after ingestion of wild mushrooms. Initial contrast-enhanced CT suggested adhesional SBO. After failure of conservative management, exploratory laparoscopy identified an intraluminal mass in the distal jejunum. Mini-laparotomy with enterotomy allowed extraction of a phytobezoar composed of *Craterellus cornucopioides*. Despite marked bowel congestion and equivocal conventional viability criteria, intraoperative indocyanine green (ICG) fluorescence confirmed adequate perfusion and avoided resection. Postoperative course was complicated by paralytic ileus, successfully reversed with neostigmine. The patient was discharged on postoperative day 14. The literature review retrieved one retrospective study and seven adult case reports; bezoars accounted for 5/7 cases, predominantly located at Roux-en-Y enteric anastomoses. ICG fluorescence had not been used in any previously reported case.

**Conclusion:**

Bezoars are a rare but clinically relevant cause of SBO in transplant recipients. Early surgical exploration is warranted when conservative management fails or imaging is ambiguous. Intraoperative ICG fluorescence is a useful adjunct to assess bowel viability and may avoid unnecessary resection. Individualized dietary counseling adapted to regional habits should be considered in long-term transplant follow-up. Larger contemporary series are needed to refine the etiologic spectrum and preventive strategies in this population.

## 1 Introduction

Small bowel obstruction (SBO) is a frequent surgical emergency. In transplant recipients, its etiology may differ from the general population due to immunosuppression, altered gastrointestinal anatomy, and pharmacologic effects on motility (1, 2). After liver transplantation, SBO occurs in approximately 1%–2% of recipients, with adhesions and internal hernias—particularly those associated with Roux-en-Y choledochojejunostomy—accounting for the majority of cases (2–5).

Bezoars are conglomerates of indigestible material that accumulate within the gastrointestinal tract and fail to resolve spontaneously (6). They are most commonly gastric but may occur at any segment of the gastrointestinal tract (7). Four classical types are distinguished according to composition: phytobezoars (plant fibers), trichobezoars (hair, classically associated with trichotillomania and trichophagia in patients with underlying psychiatric disorders), lactobezoars (milk products, typically in neonates), and pharmacobezoars (medications such as unabsorbed bile acid derivatives) (8). Predisposing factors include delayed gastric emptying, hypochlorhydria, prior gastric or bariatric surgery, altered foregut anatomy, poor dentition, and dietary habits involving high-fiber or poorly digestible foods (7).

Solid-organ transplant recipients may be at increased risk of bezoar formation through several convergent mechanisms: altered anatomy from reconstructive procedures (e.g., Roux-en-Y biliary or enteric drainage), motility disturbances related to immunosuppressive therapy, and microbiome alterations (9). The risk is best documented in lung transplantation for cystic fibrosis and in pancreas transplantation with enteric drainage (10). Evidence in liver transplant recipients remains limited to isolated case reports (11).

No transplant-specific dietary guidelines currently exist for bezoar prevention (12), and contemporary large-scale data on diagnostic and therapeutic strategies for SBO of unusual etiology in this population are lacking. Delayed recognition may result in bowel ischemia and avoidable resection.

The objectives of this report are threefold: (i) to describe a case of phytobezoar-induced SBO in a twice-transplanted liver recipient; (ii) to summarize, through a focused literature review, the published experience of mechanical SBO in adult transplant recipients with particular attention to bezoars; and (iii) to discuss the role of intraoperative ICG fluorescence for bowel viability assessment and the place of dietary counseling in post-transplant follow-up.

## 2 Case presentation

A 60-year-old man with a history of two orthotopic liver transplantations (2012 for chronic hepatitis C; 2022 for ischemic cholangiopathy) presented to a peripheral hospital with acute, diffuse abdominal pain and bilious vomiting beginning approximately 24 h after consumption of foraged wild mushrooms. Biliary reconstruction at the second transplantation had been performed via Roux-en-Y hepaticojejunostomy. Maintenance immunosuppression consisted of tacrolimus and mycophenolate mofetil.

On admission, the abdomen was distended and tender without peritonism. Contrast-enhanced abdominal CT demonstrated dilated small bowel loops with a transition zone in the distal jejunum and the “small bowel feces sign” (Figure 1), initially attributed to adhesional obstruction given the surgical history. Conservative management with nasogastric decompression, intravenous fluids, and bowel rest was initiated. After 48 h without clinical or radiological improvement, the patient was transferred to our tertiary center.

**Figure 1:**
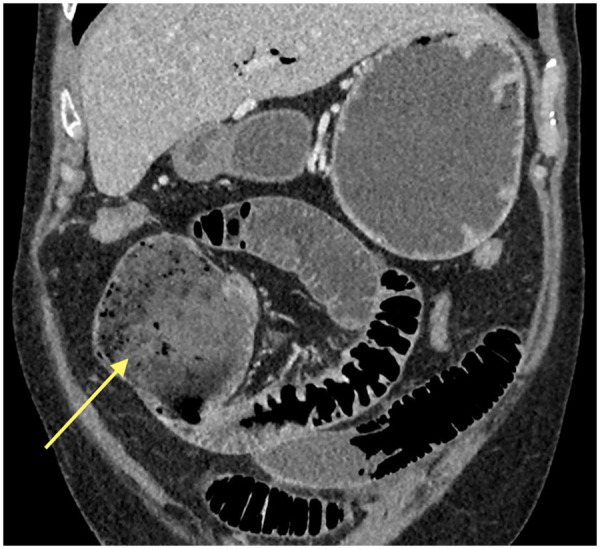
Coronal contrast-enhanced abdominal CT scan showing dilated small bowel loops with a transition zone in the distal jejunum and the “small bowel feces sign” (yellow arrow).

Diagnostic laparoscopy identified a clear transition zone with an intraluminal mass in the distal jejunum (Figure 2). A short midline mini-laparotomy was performed; enterotomy allowed extraction of an obstructing fragmented material, macroscopically composed of undigested mushroom fragments. The extracted material displayed features that were considered near-pathognomonic of *Craterellus cornucopioides* (horn of plenty, also known as “trumpet of death”): a deeply funnel- or trumpet-shaped fruiting body, a dark grey to black, shiny and shallowly wrinkled outer surface, and a thin, brittle, hollow structure, consistent with the macroscopic description reported in the mycological literature (30) (Figure 3). Combined with the patient's anamnestic context of recent wild mushroom ingestion, these features were considered sufficient for macroscopic identification by the surgical team; no formal expert mycological identification was therefore requested. Bacteriological cultures of the extracted fragments were performed and returned negative, ruling out associated bacterial contamination. The proximal jejunal loop appeared markedly congested and dusky. Conventional viability criteria were discordant: bowel color and peristalsis were altered, whereas pulsation of the mesenteric arterial branches at the mesentero-serosal junction of the small bowel was clearly preserved on direct visual inspection. Intraoperative ICG fluorescence imaging was performed using the PINPOINT® Endoscopic Fluorescence Imaging System (Novadaq Technologies ULC, Mississauga, ON, Canada; now part of Stryker Corporation, Kalamazoo, MI, USA), with standard manufacturer settings and a fixed working distance, after a 0.25 mg/kg intravenous bolus of indocyanine green. Perfusion was assessed qualitatively in real time by visual inspection of the dye distribution in the overlay fluorescence mode. Adequate arterial perfusion was defined *a priori*, based on the operating team's institutional practice, as a time-to-fluorescence of approximately 15 s or less between the appearance of fluorescence at the aorta (flushing phase) and its appearance in the small bowel wall, followed by progressive enhancement of the colon, reflecting the expected physiological inflow sequence (aorta → small bowel → colon). The affected jejunal segment met this criterion, with homogeneous fluorescence of the bowel wall reached within the predefined time window. As visual interpretation can vary between observers, ICG intensity was graded on a simple three-tier visual scale (“no fluorescence”, “low to weak fluorescence”, and “moderate to high fluorescence”) and was scored as “moderate to high”. This qualitative assessment provided immediate real-time intraoperative feedback regarding the perfusion of the affected bowel segment and supported the decision to preserve the bowel without resection. The enterotomy was closed transversely.

**Figure 2:**
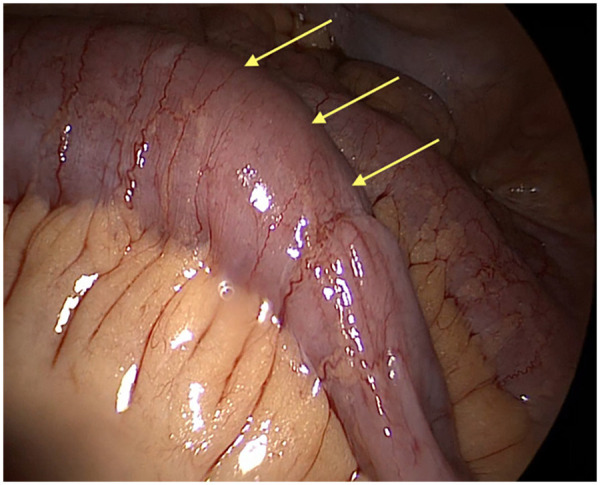
Laparoscopic view of the distal jejunum showing an abrupt transition zone (yellow arrows) with upstream dilatation.

**Figure 3:**
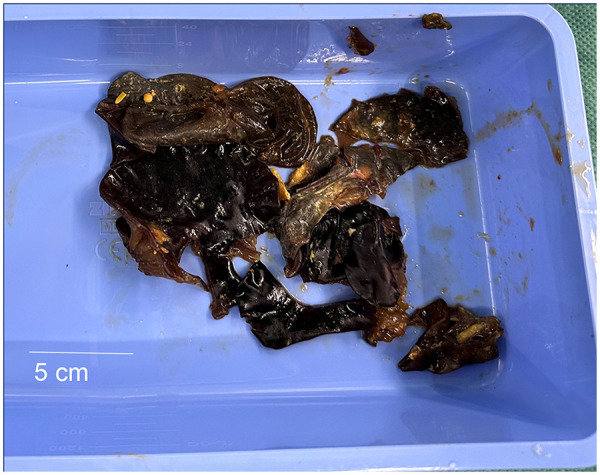
*Craterellus cornucopioides* (trumpet of death) mushroom fragments extracted through enterotomy (scale bar = 5 cm).

Postoperative recovery was complicated by prolonged paralytic ileus, successfully reversed with intravenous neostigmine. Bowel function progressively recovered, and the patient was discharged on postoperative day 14 in good general condition. The clinical timeline is summarized in Figure 4.

**Figure 4:**
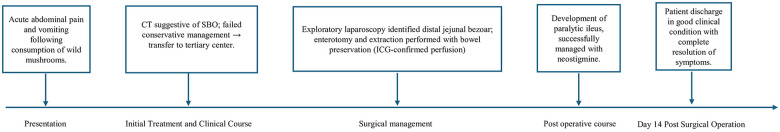
Timeline of the episode of care: presentation, initial conservative management, tertiary transfer, surgical management with ICG-confirmed viability, postoperative course, and discharge on day 14.

## 3 Literature review

### 3.1 Search strategy

A focused narrative literature review was conducted between 1st December 2025 and 31st January 2026. PubMed/MEDLINE, Embase, Cochrane Library, Google Scholar, OpenEvidence, and Litmaps were searched. Conference abstracts were not searched for the current narrative review. No restriction was applied on publication year; the cut-off date for inclusion was 31 January 2026. The following search string was applied with adaptation to each database:

(“small bowel obstruction” OR “intestinal obstruction” OR “ileus”) AND (“transplant*” OR “liver transplantation” OR “kidney transplantation” OR “pancreas transplantation” OR “lung transplantation”) AND (“bezoar” OR “phytobezoar” OR “trichobezoar” OR “pharmacobezoar” OR “enterolith” OR “foreign body” OR “gallstone ileus”).

Reference lists of included articles were manually screened for additional eligible reports.

### 3.2 Eligibility criteria

**Inclusion:** adult (≥18 years) solid-organ transplant recipients; mechanical SBO confirmed clinically, radiologically, or intraoperatively; case reports, case series, or observational studies in English with sufficient clinical detail (transplanted organ, interval since transplantation, etiology of obstruction, management, outcome).

**Exclusion:** pediatric cases; gastric outlet obstruction without small bowel involvement; bezoars without obstruction; reviews and editorials without primary data; non-English publications; reports without sufficient clinical detail.

### 3.3 Selection process

Records were screened by one reviewer (AS) with secondary verification by a second author (AK). Fifteen records were initially identified. Eleven were assessed for full-text eligibility. After application of exclusion criteria, one retrospective study (2) and seven case reports (1, 11, 13–17) were retained. The selection process is summarized in the PRISMA-style flow diagram (Supplementary Figure S1).

### 3.4 Results of the review

**Demographics and transplant context** (Table 1). Among the seven case reports (5 men, 2 women; median age 59 years, range 26–70), the transplanted organ was liver in 4 cases, simultaneous liver–kidney in 1, kidney in 1, and pancreas in 1. The interval between transplantation and SBO was highly heterogeneous (range 1 week to 240 months), with longer intervals predominantly observed after liver transplantation.

**Table 1:** Adult transplant recipients with mechanical small bowel obstruction—case reports included in the focused literature review.

Authors	Sex	Age	Organ	Predisposing factors	Interval (mo)	Signs/Symptoms	Imaging	Site	Cause	Initial Tx	Intervention	ICG	Resection	Enterotomy
Atthota et al. (13)	M	26	Liver	Roux-en-Y choledochojejunostomy	240	Abdominal pain, nausea, vomiting	CECT	Roux-en-Y	Phytobezoar	Conservative	Laparotomy	No	Yes	No
Ribeiro et al. (14)	M	67	Liver	PTLD, biliary stenosis	240	RUQ pain, jaundice, fever	ERCP, CECT	Distal jejunum	Gallstone ileus	Conservative	DBE	No	No	No
Goussous et al. (15)	M	59	Liver	Roux-en-Y hepaticojejunostomy	48	Abdominal pain, nausea, vomiting	CECT	Roux-en-Y	Foreign material	N/A	Laparotomy	No	Yes	No
Pan et al. (1)	F	65	Liver + Kidney	—	0.25	Nausea, non-bilious emesis	CECT	30 cm from ICV	Trichobezoar	Conservative	Laparotomy + extraction	No	No	Yes
Milillo et al. (16)	M	39	Kidney	—	12	Pain, constipation, distension	CT w/o contrast	Sigmoid	Phytobezoar	Surgical	Laparotomy, manual evacuation	No	No	No
Mukai et al. (11)	M	70	Liver	Cholestasis, UDCA therapy	84	Signs of pneumonia	CECT	Roux-en-Y	Pharmacobezoar (UDCA enterolith)	Endoscopic	Failed DBE → surgery	No	No	Yes
Boggi et al. (17)	F	45	Pancreas	—	0.25	Flank/RUQ pain with guarding	CECT	Roux-en-Y	Trichobezoar	Surgical	Laparotomy, fragmentation	No	No	No

CECT, contrast-enhanced CT; DBE, double-balloon enteroscopy; ICG, indocyanine green; ICV, ileocecal valve; PTLD, post-transplant lymphoproliferative disorder; RUQ, right upper quadrant; UDCA, ursodeoxycholic acid. Interval expressed in months.

**Etiology.** A bezoar was identified as the obstructing agent in 5/7 cases [3 phytobezoars (13,16 and present case equivalent), 2 trichobezoars (1, 17), 1 pharmacobezoar—an unconjugated ursodeoxycholic acid enterolith (11)]. Non-bezoar causes included gallstone ileus (*n* = 1) (14) and intraluminal foreign material (*n* = 1) (15). Notably, four of the seven obstructions occurred at or immediately distal to a Roux-en-Y enteric anastomosis (11, 13, 15, 17), consistent with anatomical narrowing as a predisposing factor.

**Diagnosis.** Contrast-enhanced CT was the diagnostic modality of choice in nearly all cases. The exact etiology was not always identified preoperatively; in one case, CT initially suggested adhesional obstruction (1), paralleling the present case.

**Management.** Initial conservative management failed in three cases. One patient was successfully treated by double-balloon enteroscopy (preferred over surgery due to comorbidities) (14). The remaining six patients required surgery: bowel resection with anastomosis (*n* = 2) (13, 15), enterotomy with extraction (*n* = 2) (1, 11), or fragmentation and milking into the stomach or cecum (*n* = 3) (14, 16, 17). Intraoperative ICG fluorescence was not used in any of the published cases, highlighting the novelty of its application in the present report.

**Outcomes.** All patients had favorable outcomes after definitive treatment. Predisposing factors—altered anatomy, prior surgery, specific dietary habits, or psychiatric history in the trichobezoar cases—were identifiable in most patients.

**Comparison with the broader literature.** The single retrospective study retrieved (2) analyzed 48 cases of SBO after liver transplantation (1985–1999) and reported adhesions (≈75%) and internal hernias (≈21%) as dominant etiologies, with no bezoar cases. This contrasts with the case-report literature in which bezoars predominate—a discrepancy likely reflecting publication bias toward unusual etiologies and the absence of a contemporary large-scale series revisiting the etiologic spectrum of post-transplant SBO in the modern era of immunosuppression and surgical reconstruction.

## 4 Discussion

### 4.1 Etiology and risk factors in the context of the present case

In adult liver transplant recipients, adhesions and internal hernias account for the vast majority of SBO (2, 3). Bezoars represent an exceptional cause but should be considered when imaging is non-specific or when clinical context suggests altered anatomy, motility disturbance, or a recent dietary trigger (6, 7).

Several features of our patient align with the predisposing factors identified in the reviewed literature. First, **anatomical alteration**: like four of the seven reviewed patients (11, 13, 15, 17), our patient had a Roux-en-Y biliary reconstruction creating a relative narrowing and altered transit. Second, **immunosuppression-related dysmotility**: tacrolimus and mycophenolate mofetil, the mainstay of modern liver transplant regimens, have been associated with delayed gastric emptying and impaired small bowel motility (9). Third, **dietary trigger**: ingestion of *Craterellus cornucopioides*, a mushroom rich in indigestible chitin and fibrous structures, has been previously reported as a cause of phytobezoar formation, alongside *Lentinus edodes* (shiitake) (18). The convergence of these three factors plausibly accounts for the present obstruction.

Notably, our case contrasts with the trichobezoar reports in the review (1, 17), in which psychiatric comorbidities (trichotillomania with trichophagia) were the principal driver—a reminder that, when bezoars are encountered, a careful psychiatric and dietary history should be obtained systematically.

### 4.2 Types of intervention in the management of bowel obstruction

All cases included in the present review were ultimately managed surgically, with the exception of one patient treated by double-balloon enteroscopy (1, 11, 13, 15–17). According to the World Society of Emergency Surgery guidelines, the management of small bowel obstruction depends on the etiology and the patient's clinical status (19). Failure of a 72 h conservative treatment trial, together with CT findings suggesting the presence of intraluminal foreign material, favors an operative approach (19, 20). Surgery can be performed either laparoscopically or via laparotomy (19, 20), and extraction of the obstructing material is generally required to relieve the obstruction (19–21).

Endoscopic management, although not generally preferred as first-line treatment in this setting, can be a valuable option in selected transplant recipients with multiple comorbidities who are not optimal surgical candidates, as illustrated by the case reported by Ribeiro et al. (14). Available evidence regarding the use of double-balloon enteroscopy (DBE) for the retrieval of small bowel foreign material remains scarce and is essentially limited to isolated case reports (22). In our view, an endoscopic-first approach may be considered when the following criteria are met: absence of clinical or radiological signs of bowel suffering, ischemia, or perforation; an accessible location of the foreign material within reach of upper endoscopy or DBE; high surgical risk related to immunosuppression or significant comorbidities; and local availability of advanced enteroscopy expertise. Conversely, surgical exploration should be favored in the presence of complete obstruction, suspected bowel ischemia or perforation, an inaccessible foreign body location, or after failure of an initial endoscopic attempt. The altered anatomy frequently encountered in liver transplant recipients with a bilioenteric anastomosis, as well as the exact location of the obstruction, must therefore be carefully assessed before opting for endoscopy (14). Disadvantages of endoscopy compared with surgery include the risk of bowel perforation, potential worsening of the obstruction, and delay of definitive treatment after repeated failed attempts (14, 19–22).

Given the very limited evidence base regarding the use of endoscopy for foreign-body-related small bowel obstruction in transplant recipients, decision-making must remain individualized and multidisciplinary, aiming to relieve the obstruction without further compromising the patient's clinical status (14).

### 4.3 ICG fluorescence for bowel viability assessment in acute obstruction

Intraoperative assessment of bowel viability remains a clinical challenge. Conventional criteria such as bowel color, peristalsis, and pulsation of the mesenteric arterial branches are subjective, with reported diagnostic accuracy as low as 50%–70% in borderline cases. ICG fluorescence offers a real-time, reproducible, and inexpensive adjunct.

Most published evidence on ICG in gastrointestinal surgery has focused on the prevention of anastomotic leak in colorectal procedures (23–26), including the recent AVOID trial (27), which did not demonstrate a statistically significant reduction in clinically relevant anastomotic leakage [relative risk 0.77 (95% CI 0.50–1.20); *p* = 0.24] but confirmed the feasibility and safety of the technique. Although these data do not directly transfer to acute SBO, they establish the reproducibility of ICG-based perfusion assessment.

The present case illustrates a distinct and, in our view, underreported application of the same technology: confirming viability of congested but potentially salvageable bowel in the setting of mechanical obstruction, thereby avoiding resection. To our knowledge, none of the seven reviewed cases used intraoperative ICG. This represents the principal novel methodological contribution of the present report. Emerging artificial-intelligence-based quantitative analysis of ICG kinetics (28) may further reduce inter-observer variability and warrant prospective evaluation in this indication.

Several important limitations of ICG fluorescence must be acknowledged before its standardized implementation in bowel viability assessment (23–26). Qualitative assessment, although fast and easy to use, is highly subjective, is subject to inter-observer variability, and cannot provide validated cut-off perfusion values (23–26). External factors such as camera distance, ICG dosage, ambient lighting and imaging settings further contribute to this variability, and should ideally be standardized within institutional protocols (23). Qualitative ICG also has a limited ability to discriminate venous congestion from arterial insufficiency in segments showing absent or low fluorescence, which may lead to unnecessary resection (23–26). Conversely, misinterpretation of preserved venous flow in early or purely venous ischemia could provide false reassurance and result in postoperative bowel ischemia (23–26). To date, no internationally validated time-to-fluorescence cut-off has been established for bowel viability assessment, and the institutional threshold applied in the present case (approximately 15 s between aortic and small bowel wall fluorescence) should therefore be considered a pragmatic, expert-based criterion rather than an evidence-based standard. Further studies focusing on quantitative ICG parameters—such as time-to-peak, slope, and normalized fluorescence intensity—are needed to standardize decision-making and minimize both ischemia-related complications and unnecessary bowel resection (23–26).

### 4.4 Dietary counseling and prevention

Nutritional counseling after solid-organ transplantation focuses primarily on food safety (avoidance of unpasteurized products, undercooked meats) and metabolic optimization. Recommendations specifically aimed at bezoar prevention—avoidance of poorly digestible high-fiber foods, thorough mastication, attention to dentition—are not currently embedded in standard transplant follow-up programs (12). Given the geographic and cultural variability of dietary habits [e.g., wild mushroom foraging in continental Europe, shiitake consumption during Lunar New Year in East Asia (18, 29)], individualized advice adapted to each patient's context appears more realistic than uniform restrictions.

### 4.5 Practical implications

Three practical messages emerge from this case and review:

1. In transplant recipients with SBO and equivocal imaging, early surgical exploration should be considered when conservative management fails after 24–48 h (20), given the risk of overlooked atypical etiology.
2. Intraoperative ICG fluorescence is a useful adjunct to assess bowel viability and may avoid unnecessary resection in borderline ischemic segments.
3. Targeted dietary counseling integrating regional eating patterns should be incorporated into long-term transplant follow-up.

### 4.6 Limitations

This work has several limitations. The literature review is narrative rather than systematic, was not prospectively registered (e.g., PROSPERO), and was conducted by a single reviewer with secondary verification rather than by independent duplicate screening. The number of eligible cases (*n* = 7) and the heterogeneity of transplanted organs and time intervals limit generalizability. Conference abstracts were not searched for the current literature review, which poses a potential limitation for underrecognizing similar cases. Publication bias toward unusual etiologies likely overestimates the relative frequency of bezoars compared with adhesions and hernias in real-world transplant populations. Finally, the single retrospective study available (2) dates from 1985 to 1999 and its findings may not fully reflect current transplant practice, immunosuppressive regimens, or surgical reconstructive techniques.

## 5 Conclusion

Bezoars are a rare but clinically significant cause of SBO in solid-organ transplant recipients, particularly when anatomical, pharmacologic, and dietary risk factors converge. Diagnosis is challenging due to non-specific imaging, and early surgical exploration should be considered in ambiguous presentations. Intraoperative ICG fluorescence appears valuable to confirm bowel viability and avoid unnecessary resection, an application that—based on the present review—has not previously been reported in this setting. Personalized dietary counseling integrating regional eating habits should be considered as part of long-term transplant follow-up. Larger contemporary series are needed to clarify the true etiologic spectrum of post-transplant SBO and to establish evidence-based preventive strategies.

## Data Availability

The raw data supporting the conclusions of this article will be made available by the authors, without undue reservation.
