# Supplementary material for: Unusual cause of small bowel obstruction in a liver transplant recipient: a case report and focused literature review
Source: Front Surg. 2026 Jun 8;13:1824834. doi: 10.3389/fsurg.2026.1824834 (PMC13284107; doi:10.3389/fsurg.2026.1824834)
Supplement: Supplementary file 1 [file Image1.pdf]

Supplementary Figure S1. Flow diagram of the literature search and selection

Focused narrative literature review — adult transplant recipients with mechanical small bowel obstruction (up to 31 January 2026)

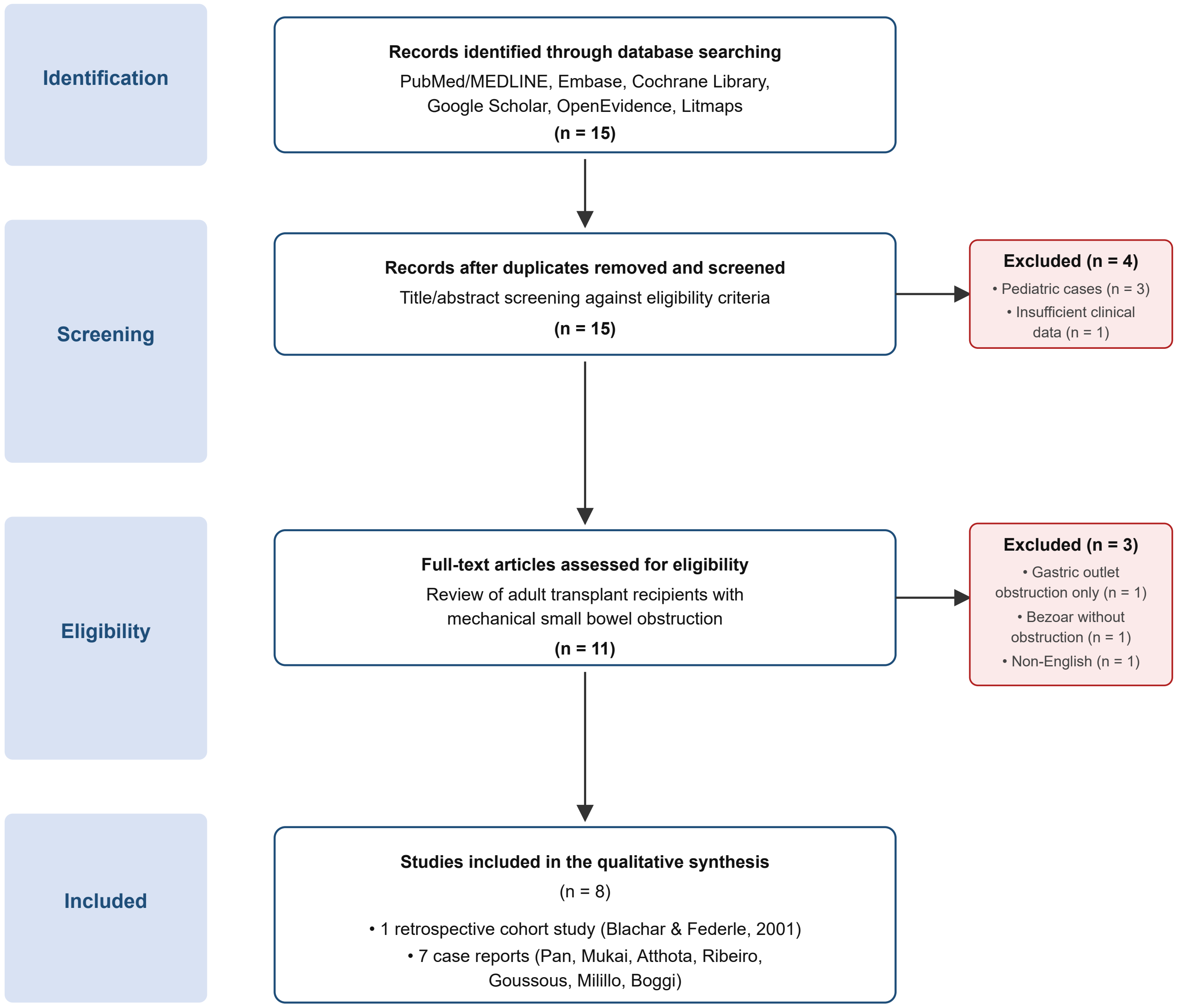

Adapted from the PRISMA 2020 flow diagram (Page MJ et al., BMJ 2021;372:n71).

Screening performed by one reviewer (AS) with secondary verification by a second author (AK).

Search string (adapted per database):

("small bowel obstruction" OR "intestinal obstruction" OR "ileus") AND ("transplant*" OR "liver transplantation" OR "kidney transplantation" OR "pancreas transplantation" OR "lung transplantation") AND ("bezoar" OR "phytobezoar" OR "trichobezoar" OR "pharmacobezoar" OR "enterolith" OR "foreign body" OR "gallstone ileus")

Period of searches: December 2025 – January 2026. Cut-off for inclusion: 31 January 2026. No restriction on publication year.
